# Significance of a neglected tropical disease: lessons from a paradigmatic case of ‘success in translation’

**DOI:** 10.1590/0074-02760200277

**Published:** 2022-04-15

**Authors:** Carlos M Morel

**Affiliations:** 1 Fundação Oswaldo Cruz-Fiocruz, Centro de Desenvolvimento Tecnológico em Saúde, Rio de Janeiro, RJ, Brasil

**Keywords:** Chagas disease, neglected tropical diseases, Innovative Developing Countries (IDCs), Trypanosoma cruzi, translational science, Southern Cone

## Abstract

In a previous publication, I stressed the fundamental importance of research for improving health using as an example the control of Chagas disease in the Americas.^(1)^ For that purpose, I analysed the major scientific breakthroughs and public health events from the 1909 discovery of Chagas disease and its causative pathogen, *Trypanosoma cruzi*, by Carlos Chagas,^(2)^ through the successful control of its transmission by insect vectors in large regions of the Southern Cone countries in the 90s.^(3)^ In the twenty years since that publication, Brazil and Latin American countries had to cope with a number of serious public health threats, old and new: (i) recrudescence of well-known diseases, such as dengue and yellow fever; (ii) emergence of viral diseases that had been restricted to other continents (Zika, Chikungunya); (iii) new epidemics (H1N1) or (iv) pandemics (COVID-19). Are there still some lessons from that success story against a neglected disease of the 90s that would be relevant today in the context of these recent challenges?

In a previous publication, I stressed the fundamental importance of research for improving health using as an example the control of Chagas disease in the Americas.^1^ For that purpose, I analysed the major scientific breakthroughs and public health events from the 1909 discovery of Chagas disease and its causative pathogen, *Trypanosoma cruzi*, by Carlos Chagas,^2^ through the successful control of its transmission by insect vectors in large regions of the Southern Cone countries in the 90s.^3^

In the twenty years since that publication, Brazil and Latin American countries had to cope with a number of serious public health threats, old and new: (i) recrudescence of well-known diseases, such as dengue and yellow fever; (ii) emergence of viral diseases that had been restricted to other continents (Zika, Chikungunya); (iii) new epidemics (H1N1) or (iv) pandemics (COVID-19).

Are there still some lessons from that success story against a neglected disease of the 90s that would be relevant today in the context of these recent challenges?

**Neglected diseases: neglected by whom? Who suffers from them?**

The concept of “neglected diseases” has evolved since the term was first used by Kenneth Warren when launching the Rockefeller Foundation program The Great Neglected Diseases of Mankind.^4^ Ken pointed his finger towards research funding agencies, such as the NIH, showing the contrast between the amount of funds committed to research in cancer versus those in parasitic diseases such as malaria or schistosomiasis.^5^ In 1999, the Médecins Sans Frontières (MSF) won the Nobel Peace Prize and decided to create the Drugs for Neglected Diseases Initiative (DND*i*), moving the concept of the phrase towards “neglected by the pharmaceutical industry”. Nowadays, the World Health Organization (WHO) refers to them as:

*Neglected tropical diseases (NTDs)* - A diverse group of communicable diseases that prevail in tropical and subtropical conditions in 149 countries - affect more than one billion people and cost developing economies billions of dollars every year. Populations living in poverty, without adequate sanitation and in close contact with infectious vectors and domestic animals and livestock are those worst affected (https://www.who.int/neglected_diseases/diseases/en/).

Two additional concepts are worth mentioning: (i) Poverty promoting diseases, implying that NTDs are, simultaneously, a consequence of poverty and a major underlying cause of economic underdevelopment;^6^ ^,^ ^7^ ^,^ ^8^ (ii) Diseases of neglected populations, which emphasises the link of these diseases to those living in poverty.^9^

*Neglected diseases and Innovative Developing Countries (IDCs)* - Since 2005, together with Richard T Mahoney, Chad Gardner, Alexandre Vasconcellos and other colleagues, we have championed the concept of IDCs:^10^ ^,^ ^11^ ^,^ ^12^ ^,^ ^13^

“All developing countries can undertake health innovation to varying degrees. Some developing countries, however, are more scientifically advanced than others and are starting to reap benefits from decades of investments in education, health research infrastructure, and manufacturing capacity. We refer to these as innovative developing countries (IDCs)”.^11^

The approaches implemented by countries to address the social and economic impact of NTDs differ substantially depending whether they are developed/industrialised, an IDC or a Least Developed Country (LDC): (i) Developed countries are in general free from Most Neglected Diseases (Type III) and have the resources and interventions to prevent and control those Type II Neglected Diseases that are still present in their populations such as tuberculosis (for the classification of diseases into Global or Type I, Neglected or Type II, and Most Neglected or Type III see.^14^ ^,^ ^15^ For these reasons NTDs are not regarded as “high priority” by their governments, health systems and services; (ii) Innovative Developing Countries, IDCs, are in general aware that NTDs should represent a priority public health issue: In fact, a recent study demonstrated that IDCs rank among the top countries according to the proportion of their scientific publications addressing at least one NTD;^13^ (iii) Least Developed Countries, LDCs, which suffer the highest toll from the burden of NTDs (Neglected/Type II and Most Neglected/Type III), lack the infrastructure and the human and financial resources needed to prevent and control those diseases. Most LDCs, therefore, look for external help, support and financial means to deal with NTDs.

Based on these definitions, the IDCs occupy a special niche - suffering the burden of NTDs and having the means to prevent, mitigate or control them - which rightly impose moral, ethical and human rights responsibilities on their governments, decision makers and public health systems to commit resources to address endemic NTDs that also has a potential to benefit LDCs.

*Translational science and the valley of feath* - Establishment of the concept, “translational science”, as a major field of activity in biological and biomedical sciences, along with its strengthening over the past couple of decades, can be traced to a few significant initiatives and facts: (i) In 2006, the National Institutes of Health (NIH) established the Clinical and Translational Science Awards (CTSA) Program, recognizing the need for a new impetus to encourage clinical and translational research. At the time, it was challenging to translate basic and clinical research into clinical and community practice; making it difficult for individual patients and communities to receive its benefits (https://www.nap.edu/read/18323/chapter/1);^16^ (ii) In two seminal articles published in 2007-8 by Declan Butler, Nature’s European correspondent based in France and its senior reporter, the existence of a critical gap was identified between laboratory research progress and the delivery of tools and interventions to those most in need:

- The culture of academia needs to change if scientists are to bridge the gap between research and the development of drugs and vaccines for neglected diseases in the developing world (article ‘Lost in translation’, 2007”;^17^

- A chasm has opened up between biomedical researchers and the patients who need their discoveries (article ‘Crossing the valley of death’, 2008);^18^

- In October 2009, the American Association for the Advancement of Science launched the journal Science Translational Medicine;^19^

- In December 2011, the United States started the National Centre for Advancing Translational Sciences (NCATS) program.^16^

This movement faced hard criticism at its beginning for proposing a continuum from basic research into applied sciences, technological development and health innovation, which was a major departure from the traditional ‘linear model’ of product development that separated basic research (performed in universities) from applied research (restricted to industry).^20^ According to Reinaldo Guimarães, “Translational Research interpreta a demanda de uma indústria farmacêutica poderosa e em crise, associada a dificuldades de outputs científicos em quantidade adequada para atendê-la como um móvel suficiente para explicar a intervencão do governo norte-americano na pesquisa biomédica através do NIH.”^21^

Today, translational science has become a regular, more routine approach to plan and manage biomedical programs that address a wide spectrum of activities: basic research, preclinical research, clinical research, clinical implementation, public health including patient involvement (https://ncats.nih.gov/translation/spectrum).^22^ Due to this expansive range of these activities, today’s metrics of success are no longer limited to those classical indicators at the beginning and end of the R&D spectrum (number of scientific publications or of goods produced), but are spread along all the several areas of work of Translational Research Organizations (TROs): funding, talent, creation, validation, dissemination, external uptake, collaboration.^23^

A clear demonstration of the acceptance of the translational science framework is the success of NCATS and product development partnerships (PDPs), not-for-profit organisations that adopt good translational science practices to link academia, industry and donors to generate and deliver new tools and interventions for those who need most. In the long journey trying to develop new tools and interventions against NTDs, these organisations have succeeded in collecting a few success stories that bridge and cross some valleys of death (examples of PDPs: Medicines for Malaria Venture (MMV https://www.mmv.org/), established in 1999; The Global Alliance for Tuberculosis Drug Development (TB Alliance https://www.tballiance.org/), established 2000; Drugs for Neglected Diseases initiative (DNDi https://www.dndi.org/) and the Foundation for Innovative New Diagnostics (FIND https://www.finddx.org/), both established in 2003.

**Chagas disease: a case of ‘success in translation’?**

On the centenary of the discovery of Chagas disease, Kropf^24^ and Carvalheiro et al.^25^ described the historical work of the scientific pioneers responsible for the discovery and research studies that established Chagas disease as a major scourge of mankind and one of the NTDs most afflicting Latin America.

In this article, I call particular attention to success stories and breakthroughs in the last decades that should teach us a broader lesson: some IDCs, such as Brazil, have the human resources and infrastructure that empower their research and development programs to be at the forefront of prevention and control of not only NTDs, but also other immediate public health challenges, such as epidemics and pandemics, especially those declared a Public Health Emergency of International Concern (PHEIC).^26^

*The pillars of a successful policy* - From a historical perspective, it is important to highlight: (i) the long and complex pathway that led to the recognition of Chagas disease as both a scientific fact and a public health problem in Brazil;^27^ (ii) that the idealisation, organisation and management of the Southern Cone initiative only became possible because of previous scientific, technological and organisational breakthroughs (full details in^1^):

- 1947: Field trials conducted by Dias and Pellegrino in Brazil and Romaña and Abalos in Argentina demonstrating the efficacy of organochlorine insecticides against domiciliated triatomine bugs;

- 1950: Creation of the first National Control Programmes in Brazil and Argentine;

- 1974: CNPq launched the Programa Integrado de Doenças Endêmicas (PIDE) and started the Annual Meetings on Basic Research in Chagas Disease in Caxambu, Minas Gerais, Brazil;

- 1975: Establishment of the UNDP/World Bank/WHO Special Programme for Research and Training in Tropical Diseases (TDR), hosted by the World Health Organization in Geneva, which included Chagas disease in its priority portfolio of NTDs;

- 1975-1980: Execution of national serologic and entomological surveys in Brazil;

- 1977-1979: Establishment of Chagas disease control as a national priority in Brazil.

**S&T capacity building for NTDs: the basis for addressing PHEICs**

In the 80s’ and 90s’, decades long-term investments in research, capacity building and training in NTDs by national and international agencies and organisations helped Brazil build an internationally recognised R&D community. The attendance and participation at the annual meetings in Caxambu provided an indication of its growth from 30 abstracts presented in 1975 that grew steadily to 666 abstracts in 1998.^1^ This evolution was not only in quantity but also in quality, as described in two ‘news and views’ articles published in international, peer-reviewed scientific journals describing results first presented at these annual Caxambu meetings on Basic Research in Chagas Disease:

- Caxambu, Brazil - When hundreds of biologists from Brazil and elsewhere gathered here for a recent meeting, the work they discussed would not have been out of place at Cold Spring Harbor or Heidelberg: genome mapping and sequencing, protein crystallography, and rational drug design. But all this up-to-the-minute science was aimed at a traditional focus of Brazilian biology: *Trypanosoma cruzi*, the insect-borne parasite that causes Chagas disease;^28^

- Plus surprenant, deux groupes de chercheurs ont récemment conclu que ce proces- sus est à l’oeuvre dans la maladie de Chagas, une affection parasitaire qui sévit en Amérique du Sud. L’annonce en a été faite à la réunion annuelle de recherche fondamentale sur la maladie, qui s’est tenue en novembre dernier dans la petite ville de Caxambu, dans le sud-est du Brésil.^29^

A ‘double miracle’ had occurred: the long-term investments in training and capacity building for R&D in NTDs that lead to the successful control of Chagas disease had also created a strong, mature and vibrant scientific community. How would it react to future challenges?

**Talent spillover: how countries addressed epidemics**

‘Spillover’ is a term often used by researchers and writers to describe the events that lead a zoonotic virus to jump from its wild reservoir to infect a human host.^30^ Here, I propose to repurpose the word to another meaning that captures the potential of a highly trained scientific community to securely embrace their abilities to spillover its expertise to address demands in other areas, pressed or not by emergency needs (Do not give me fish, teach me how to fish instead would be another way to illustrate the concept of ‘knowledge or talent spillover’).

In a recent article, together with Vasconcellos and Fonseca, we analysed two sanitary crises that impacted neglected populations in Africa and South America between 2014-2016: the Ebola epidemics in West Africa and the spread of Zika virus in South America:^13^

- The majority of the work conducted in West Africa to detect, diagnose and control the Ebola epidemics was carried out by teams brought from abroad in response to a dramatic appeal from the Director General of the World Health Organization when she declared the Ebola epidemics a Public Health Emergency of International Concern (PHEIC). On that occasion, it was emphasised that West African countries’ health systems needed international help to manage infection.

- The Zika epidemics in the Americas that started in the northeast of Brazil, on the other hand, was detected and characterized by physicians and researchers working at local health services, hospitals or universities. Brazilian scientists were responsible for seminal work on outbreak characterization, clinical case definition, sexual trans- mission. Furthermore, their research was critical to document the anomalous high incidence of microcephaly and other newborn malformations that associated them with Zika virus infecting pregnant women, and precipitated studies on antiviral treatment and vector biology.

The importance of the work of local institutions, researchers and policy makers working at the epicentre of the Zika epidemics was recognised by the international journal Nature when it distinguished Dr Celina Turchi, from the Aggeu Magalhães Research Institute, an institution affiliated to the Oswaldo Cruz Foundation in Recife, State of Pernambuco, Brazil as one of the journal’s 2016 ‘top-ten’ scientists.^31^ The prominence of the ‘Aggeu’ Institute during the Zika epidemics can only be understood taking into consideration the long-term investments into building its capacity by national and international organisations. In the 80s’ and 90s’ Fiocruz and TDR invested massively in the Aggeu Magalhães Institute in all aspects: infrastructure, exchange of scientists and human resources training in NTDs, with a focus on lymphatic filariasis, a major scourge in Recife. Construction of new headquarters and facilities, moving the institution from its old premises to a complex of new buildings inside the campus of the Federal University of Pernambuco, UFPE, represented a radical upgrade of this Fiocruz regional research centre.^32^ In a previous publication, we called to attention the importance of the pre-existing healthcare infrastructure and research networks in an IDC to mount an effective response against an emerging health threat. The overall response to the Ebola epidemic, which only affected the least developed countries, was primarily driven by outside experts who were severely constrained by local customs and societal norms.^13^ In these LDCs, there was an absence of resident talents trained within the affected the countries to ‘spillover’.

*In Conclusion* - Recognising the advances and victories leading to Chagas disease control in the Southern Cone made me classify these events and the present-day situation as a paradigmatic case of Success in translation, as stated in the title of this article. This success story is the result of a long process led by incredible people, each one a leader in her or his area of work, who were able to collaborate with equally dedicated partners at the decision-making and political levels. A global victory for several countries and their leadership that has been praised in various major health and science events.

Unfortunately, my final words at this moment are not of optimism, as the present-day situation and global scenarios are quite different. It is impossible not to be reminded of the words of Lewis Carroll in his book, “Alice through the looking glass”: now, here, you see, it takes all the running you can do, to keep in the same place. If you want to get somewhere else, you must run at least twice as fast as that!

Instead of running twice as fast, powerful forces of ignorance, stupidity, prejudice and hatred are already undermining the favourable atmosphere of the last decades. The beautiful sagas we are proud of today, might soon be only a chapter in history books. Major cuts in health and S&T budgets, radical cuts and decrease in the support to train young graduate students along with the destruction of Brazil’s major S&T agencies CNPq, FINEP and CAPES, represent a catastrophe that will impact our country potentialities during decades.
